# The people behind the pounds: a qualitative exploration of factors that help or hinder healthy, sustainable food purchases for people living with obesity and food insecurity in the UK

**DOI:** 10.3389/fnut.2025.1646056

**Published:** 2025-10-08

**Authors:** Emma Hunter, Alexandra M. Johnstone, Rebecca A. Stone, Hannah C. Greatwood, Charlotte A. Hardman, Adrian Brown, Claire Griffiths, Flora Douglas

**Affiliations:** ^1^School of Health, Robert Gordon University, Aberdeen, United Kingdom; ^2^The Rowett Institute, University of Aberdeen, Aberdeen, United Kingdom; ^3^Department of Psychology, University of Liverpool, Liverpool, United Kingdom; ^4^School of Health, Leeds Beckett University, Leeds, United Kingdom; ^5^The Obesity Institute, Leeds Beckett University, Leeds, United Kingdom; ^6^Centre for Obesity Research, University College London, London, United Kingdom

**Keywords:** food insecurity, obesity, supermarkets, lived experience, cost-of-living, health inequalities, qualitative research

## Abstract

Good health is viewed as essential to enable citizens to live fulfilling lives, shape communities, and drive economic growth. However, health is socially patterned. Low socioeconomic status is associated with an increased risk of non-communicable diseases, where poor dietary patterns and diet-related obesity are likely contributors. Food purchasing can be influenced by many factors, including cost and income. Most food purchased to be consumed at home is acquired from supermarkets, and any increase in food prices disproportionately impacts low-income households, contributing to food insecurity. This study explored the factors that helped and hindered people living with obesity and food insecurity in purchasing healthy, environmentally sustainable food from supermarkets. Semi-structured interviews (*n* = 25) and focus groups (*n* = 7) were conducted between June and December 2023 with adults living in Scotland and England who self-identified as living with obesity and food insecurity. Using thematic analysis, six main themes were identified: (1) *Supermarket deals*: perceptions surrounding *the good*, *the bad,* and *the ugly* side of supermarket offers and promotions; (2) *Skepticism about supermarkets and the wider food system*: questioning supermarket pricing motives but recognizing the role of the wider food system in food pricing; (3) *Other peoples’ role in enhancing or undermining healthy diet intentions*: the impact of others in shaping food purchases; (4) *Financial restrictions facing non-UK nationals*: additional challenges faced by those with no recourse to public funds; (5) *The overwhelming in-store supermarket experience*: sensory overload and attempts to prevent unintended, impulse purchases; (6) *Unconscious, environmentally sustainable shopping practices*: cost saving practices that lead to environmentally sustainable purchasing patterns and behaviors as a unintentionally created outcome of budget maximizing strategies. However, such strategies, that is, limiting food waste and purchasing less meat, although beneficial for environmental sustainability, do not necessarily indicate that a healthier diet is being purchased or consumed. While views on some factors believed to help or hinder healthy, environmentally sustainable food purchases varied, there was general agreement amongst participants on the need for upstream changes, including having access to adequate benefits and wages.

## 1 Introduction

Good health is defined as ‘a structural, functional, and emotional state that is compatible with effective life as an individual and as a member of society’ (1). Good health can be viewed as essential in enabling people to live life to the full, shape communities, and drive economic growth (2). However, health is socially patterned, including in a developed country such as the UK. Low socioeconomic status is associated with an increased risk of non-communicable diseases, including cardiovascular disease and cancer (3–5), and shortened life expectancy (6–8). Poor dietary patterns, such as those high in refined carbohydrates, red and processed meats, and lacking fruit, vegetables, and whole grains (5, 7, 9, 10), and, in turn, diet-related obesity (11), are all likely contributing factors to such diseases and shorter lifespan.

A healthy diet is essential for optimal health across the lifespan (12). To help inform their citizens about what constitutes a healthy diet to support and promote population health, Governments utilize food-based dietary guidelines (FBDGs), which are evidence-based, national food and nutrient recommendations (13), such as the UK Eatwell Guide (14). Furthermore, consuming food that aligns with UK Eatwell Guide recommendations has the potential to promote planetary health through the reduction of greenhouse gas emissions (GHGE) (15). The current food system accounts for approximately a third of GHGE, mostly through agriculture and land use, as well as supply chain activities (16). Changes to the current food system will play a vital role in the UK Government’s commitments to reach net zero by 2050 (17). While encouraging citizens to eat in line with FBGRs could support the purchase and consumption of both a healthier and a more environmentally sustainable diet, such foods tend to be more expensive than less healthy alternatives (18–20). Therefore, despite knowledge around what constitutes a healthy, sustainable diet and aspirations to eat in line with this knowledge, cheaper, high-energy-dense, less nutritious food may become the most affordable option for low-income households (21, 22) and present challenges for weight management (19, 23). Indeed, data show that in the UK, those living in the most deprived areas are more likely to be living with obesity compared to those in the least deprived (24, 25). The ability to purchase and consume a healthy, nutritious diet has become especially challenging for many in recent years.

COVID-19, the war in Ukraine, and a high demand for goods in the face of supply chain disruptions saw UK inflation rates reach a 41-year high of 11.1% in 2022 (26). The high price of food and energy, coupled with stagnating wages and benefits, resulted in the cost-of-living crisis (27, 28). There has long been a disparity between the cost of healthy and less healthy foods, as defined by the UK Government Nutrient Profiling Model (29, 30). However, between 2022 and 2024, this price gap increased substantially; healthy food prices rose by 21%, while the price of less healthy food increased by 11% (30). Consequently, the number of UK households reporting food insecurity (FI), defined as a lack of access to sufficient, good-quality, nutritious food (31), rose from 8% in 2020 to 12% in 2023 (32). To eat in line with the FBDGs set out in the Eatwell Guide, it is estimated that those in the most deprived fifth of the population would need to spend 45% of their disposable income, whereas those in the least deprived fifth would need to spend 11% of their disposable income (30). Such stark differences in the proportion of spending requirements highlight existing inequalities that contribute to socially patterned health outcomes. Research has shown that certain households, i.e., those with children, individuals living with a disability, and those from minority ethnic backgrounds, may be at increased risk of experiencing FI due to structural and socio-economic disadvantages (32–35).

The cost of eating in line with FBDGs is disproportionately high for low-income households with children. In the UK, households with children in the most deprived fifth of the population would need to spend approximately 70% of their disposable income to eat in line with the Eatwell Guide, compared to a spend of just 12% for families with children in the highest income quintile (30). Families with children and individuals reliant on financial assistance, including those living with a disability and those from minority ethnic backgrounds, often experience financial strain due to benefits failing to cover basic outgoings (34, 36–39). Subsequently, such populations may be more susceptible to the impact of austerity measures, which have been associated with negative impacts on physical and mental health outcomes, life expectancy, and mortality rates (36, 39).

In high-income countries, approximately 80% of all food purchased for consumption in the home is sourced from supermarkets (40, 41). When supermarket food prices rise, those living on a low income are disproportionately affected (30). There is a need to better understand the experiences of people living with obesity (PLWO) and FI when shopping for food in the supermarket to begin to understand what factors and strategies help or hinder the purchase of healthy, environmentally sustainable food within this context, including during times of economic instability such as the recent cost-of-living crisis (42).

This research was conducted as part of the Food Insecurity in People Living with Obesity (FIO Food) project, which aims to support healthy and environmentally sustainable food choices in the UK food system (43). This study expands on and further contextualizes previously reported findings from the FIO Food project (21, 42, 44). Previous studies reported that FI was associated with barriers from the food environment, including the high price of healthy food, and poorer mental health, and FI stigma was associated with poorer diet quality (44). Food-insecure individuals who stuck to a strict budget not only reported reductions in relation to food quality and quantity but also a reduction in the healthiness of the foods they purchased (21, 42). However, despite the restrictions around their shopping practices and their ability to buy and consume healthy foods (and the often-accompanied emotional toll experienced as a consequence of these restrictions), PLWO and FI described taking actions and using agency to mitigate the rising costs of food and the constraints of their limited budget, to try and eat as healthy and environmentally sustainable a diet as their budget would allow (21). Within the context of those lived experiences of PLWO and FI, the research aimed to surface factors which individuals found helped and/or hindered the purchase of healthy, environmentally sustainable food items when shopping in the supermarket for healthy, sustainable foods to meet their weight loss or weight maintenance goals.

## 2 Methods and materials

### 2.1 Participants

**Figure 1:**
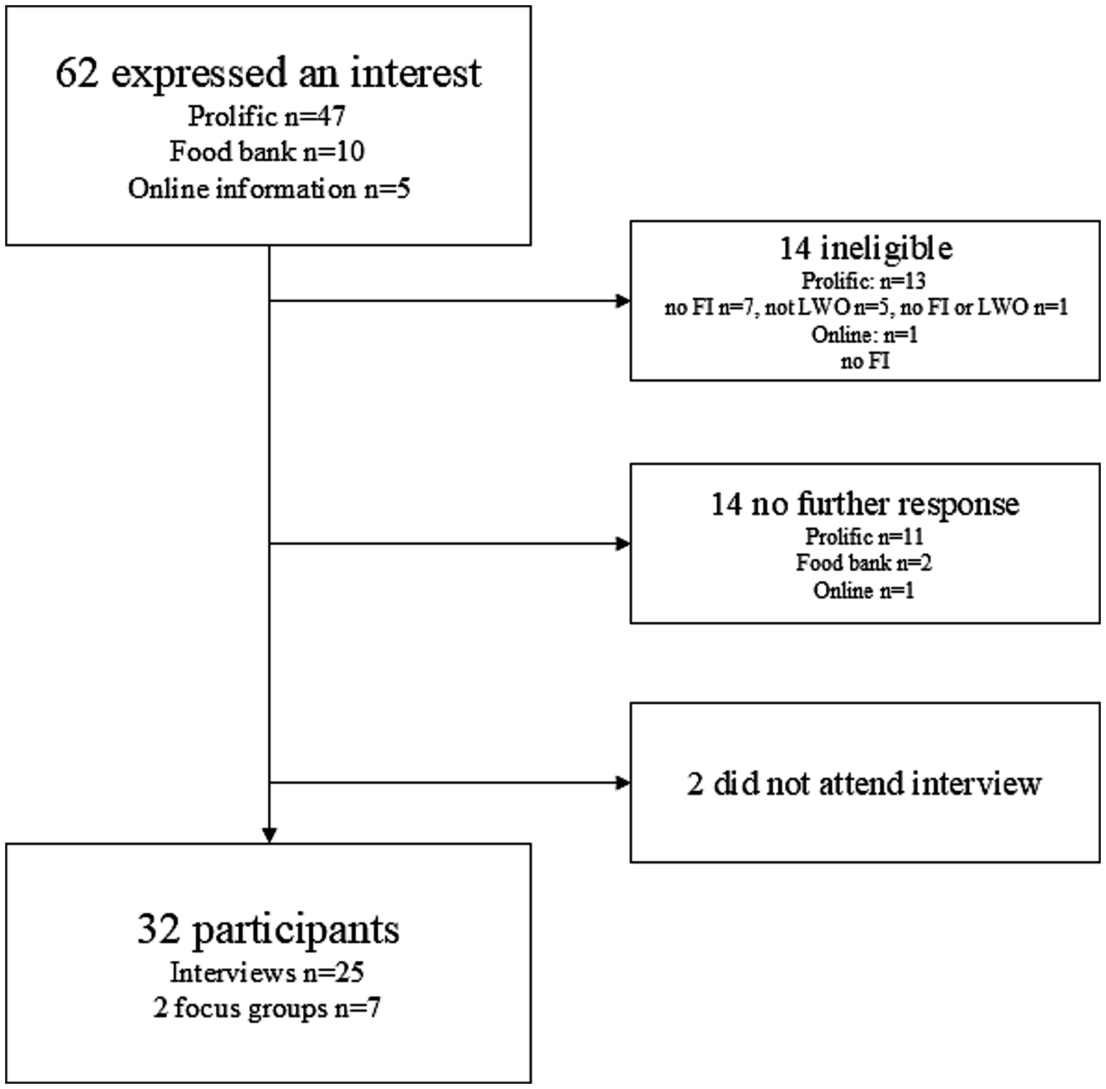
Flow diagram of participant recruitment. FI: food insecurity, LWO: living with obesity; n: number.

Participants were adults (aged over 18 years) who self-identified as both living with obesity (LWO) and FI and intending or actively taking steps to reduce their weight. Maximum variation sampling was utilized to recruit a broad and diverse range of views and experiences from individuals of different genders, ethnicities, and household sizes. Participants (*n* = 32) were recruited either through having expressed an interest following participation in a linked quantitative survey study (42) (*n* = 22), hosted on Prolific, a participant pooling website^1^, following an online press release and social media advertisements (*n* = 3), or through a food bank located in North East Scotland (*n* = 7) (Figure 1).

### 2.2 Assessing eligibility

Those who indicated an interest in taking part in the study were provided with a participant information sheet that outlined the research aims, what they would be asked to do should they agree to participate, and informed them of their right to withdraw. Potential participants were asked to complete a screening questionnaire that included a 2-item FI screener (45). This brief screening questionnaire also asked participants to self-report their height and weight, from which body mass index (BMI) was calculated. The questionnaire collected demographic information, including age, gender, ethnicity, intention or active engagement in weight reduction, and information on health conditions. Eligible participants, i.e., those experiencing FI and having a BMI > 30 kg/m^2^, were invited to an online or telephone interview or an online focus group. Those recruited through the food bank were offered the opportunity to take part in an in-person focus group at the food bank premises. Individual interviews conducted in person on the food bank premises were not possible due to the limited time available within the food bank session for discussions.

### 2.3 Informed consent and ethical approval

Recorded verbal or written informed consent to participate was sought from all eligible participants prior to the interviews and focus groups commencing. Data collection continued until data saturation was reached and no new information emerged (46). Ethical approval for the study was obtained from the Robert Gordon University, School of Nursing, Midwifery and Paramedic Practice, School Ethics Review Panel (SERP reference number 23–02, approved on 26th May 2023). Participants were recompensed with a £25 retail gift voucher for giving up their time and for sharing their experiences and expertise.

### 2.4 Patient and public involvement

Study documentation, including the 2-item FI screening questionnaire, the participant information sheet, recruitment posters, and the semi-structured topic guide (Supplementary Data), was developed in collaboration with the FIO Food project Patient and Public Involvement (PPI) partners (21). Co-production of knowledge is a fundamental principle of the FIO Food project with PPI groups established from the start (43). The PPI group also helped interpret the data and explain the study findings within their wider knowledge and experience as recommended by Brett et al. (47).

### 2.5 Data collection and analysis

Between June and December 2023, interviews were conducted online, via Microsoft Teams, or by telephone (lasting 25–50 min). Additionally, in-person focus groups (lasting 30–40 min) were conducted by E.H. Interviews and focus groups (using a TASCAM DR-07X audio recorder) were recorded by E.H and transcribed verbatim by a University-approved data transcription service (Red Balloon). All transcripts were anonymized by E.H. Field notes based on the interviewer’s observations, and other notable and relevant information were generated. All data were thematically analyzed by E.H and F.D following the steps outlined by Braun and Clarke (48). Initially, E.H and F.D familiarized themselves with the data. E.H generated initial descriptive codes that were reviewed by F.D. and discussed (E.H and F.D). Any differences of opinion and queries around any initial codes were shared with all authors during presentations and regular research team meetings. This process was continued until the themes and sub-themes were finalized. NVivo 13 software was used to manage and support data analyses.

## 3 Results

### 3.1 Participant characteristics

Participants resided in either England (*n* = 20) or Scotland (*n* = 12), were mostly women (71.9%, *n* = 23), white (65.7%, *n* = 21), and aged between 35 and 54 years (65.6%). Self-reported median BMI was 35.5 kg/m^2^. All participants self-identified as experiencing FI. Despite a high number of participants reporting their health as good or fair (71.9%, *n* = 23), just over two-thirds reported living with one health condition (68.8%, *n* = 22), with 56.3% (*n* = 18) indicating they were living with two or more. Commonly reported health conditions included type 2 diabetes, high blood pressure, arthritis, depression, and anxiety. The majority of participants were responsible for buying food for their children and/or their partner or spouse as well as themselves (65.7%, *n* = 21), and just over half of the participants (53.1%, *n* = 17) reported they had been actively attempting to reduce their weight for the past 6 months or less (Table 1).

**Table 1:** Participant demographics, food purchasing responsibility, and current dietary behavior or plans.

Characteristic	Category	*N* (%*)
Gender	Female	23 (71.9%)
	Male	9 (28.1%)
Age range (years)	18–24	2 (6.3%)
	25–34	5 (15.6%)
	35–44	13 (40.6%)
	45–54	8 (25.0%)
	55–64	2 (6.3%)
	65+	2 (6.3%)
Ethnicity	White (British, Scottish, English, Irish)	19 (59.4%)
	White (other)	2 (6.3%)
	Black	4 (12.5%)
	White and Black African	1 (3.1%)
	White and Black Caribbean	3 (9.4%)
	Mixed/ multiple ethnicity	1 (3.1%)
	Asian	1 (3.1%)
	Pakistani	1 (3.1%)
Food purchasing responsibility	Themselves only	8 (25%)
	Themselves and their partner	6 (18.8%)
	Themselves and their child/ children	6 (18.8%)
	Themselves, their partner, and their child/children	9 (28.1%)
	Themselves and the parent/ guardian	2 (6.3%)
	Themselves and a friend	1 (3.1%)
Intending/actively attempting weight reduction	Intending to reduce their weight in the next 6 months	8 (25%)
	Intending to reduce their weight in the next 30 days	3 (9.4%)
	Actively attempting to reduce their weight for < 6 months	17 (53.1%)
	Actively attempting to reduce their weight for> 6 months	4 (12.5%)

*% reported may not add to 100% due to rounding up.

### 3.2 Outline of themes and sub-themes

Our thematic analysis of the data, focusing on participants’ experiences of what helps and hinders the purchase of healthy, sustainable food when shopping in the supermarket setting, revealed six main themes and 16 associated sub-themes (summarized in Tables 2, 3). The first five themes were described and outwardly discussed by participants during the interviews and focus groups (Table 2).

**Table 2:** Main theme, sub-theme, and example quotation.

Theme	Sub-theme	Example quotation
Supermarket deals	(i) The good	‘the things that I particularly like are, in Morrisons, you can get a wonky veg box. I do not care what the carrots and leeks and whatever, I really do not care what they look like …One that I got recently was the wonky sweet potatoes. Fantastic, I got something like eight sweet potatoes for under a pound, and that’s great, I can make soup, I can make wedges, I can make mash, I can do, put them in casseroles. I do not care what they look like.’ Participant 54 (woman, age range 45–54)
	(ii) The bad	‘I try, but it does not really get me anywhere. Because, you know, I might do it 1 week when something is in, something that is healthier, is on offer and then that’ll be for 4 weeks. And then by the time that is not on offer anymore, there’s no way…I could do it for 4 weeks, 4 weeks is not gonna’ have, is no, it’s that drop in the ocean you know, so it’s just pointless. So in the end it’s like it’s like it’s just close your eyes, buy what you can afford and carry on.’ Participant 02 (woman, age range 45–54)
	(iii) The ugly	‘I very rarely see deals when it comes to fruit and veg. Erm, I think sometimes you can get a deal where you do like the stir fry thing where you get your meat and you get the noodles and things like that, so I guess that would be like a multi-buy thing, but I do not really see it on anything else, it’s always like your Cadbury’s, or Coke-a-Cola or, you know, buy one get one free on pizzas, you know, the frozen pizzas or whatever it might be, so yeah, I definitely think that they target that, it’s not here, have some nutritious flax seed, you know…It’s never the staples, is it, it’s always the junk they are trying to sell you.’ Participant 35 (woman, age range 35–44)
Skepticism about supermarkets and wider food system	(i) Skepticism in the aisles: disingenuous discounts and deals	‘you know like the Clubcard, with Tesco they do the Clubcard prices… Asda Rewards they kind of do these star products and things but you never feel that, you always feel like you are just paying the original price anyway and they have just made it look cheaper.’ Participant 44 (man, age range 35–44)
	(ii) Supermarkets are only part of the problem	‘it is not always on the supermarkets, you know, it is the state of the whole, you know, cause obviously, if there is like this energy crisis the farmer has to pay much more for the electricity, for their water, you know’ Participant 12 (woman, age range 45–54)
	(iii) Upstream change is required	‘They’ve [government] got to do something about it because the supermarkets will find a way out, they have got lawyers that will find little tiny loopholes in, even though in, erm, you know, the, the stuff that comes out of the government, they can find ways around it, but hopefully then they’ll be penalized. But you know for, to make sure, hopefully that it happens and it happens properly, it needs to be done from on high’ Participant 02 (women, age range 45–54)
Other peoples’ role in enhancing or undermining healthy diet intentions	(i) Steered toward healthy purchases	‘when I go by myself I’m much more likely to go toward like the frozen food section or like to buy like prepackaged food which I can just, you know, put in the oven or microwave and just make, but when she [sister] comes along she tends, we tend to have more of an effort to go toward like the fresher food aisle. So, for example, like chicken breast in Tesco, they have like a halal section so we tend to go there whenever, when she comes along so we can buy stuff from there and that probably does have an effect like I’m less likely to then go to the frozen food sections because we are buying these foods instead whenever I go with her. So, yeah, no, I would say there’s a difference in decision making for sure, to an extent.’ Participant 10 (man, age range 18–24)
	(ii) Decreased healthfulness and sacrificing healthy eating aspirations	‘they are [teenage children] quite fussy eaters, to tell you the truth. So, a lot of the time I’m getting stuff, like they like frozen pizzas like and it’s not helping with me weight and stuff like that.’ Participant 46 (man, age range 45–54)
Financial restrictions facing non-UK nationals	(i) No recourse to public funds	‘let me tell you one of the struggles of people of our own descent face here, most of our visa carries an exemption, and what is that exemption, not entitled to public funds, and that places a huge financial burden on us immigrants specifically. Now, every other person that is a citizen, or has whatever status, can benefit from all those things, all those government interventions, but we cannot because we are not entitled to public funds. And why we are not, I cannot see because, I’ll use myself as an example, I pay my tax, right, I live in the country legally, I was employed by an organization with a 5 year visa sponsorship, right. I pay for NHS, I mean, this is about £3,000, over £3,000 that we paid for NH, I mean for a period of that 5 years’. Participant 57 (man, age range 35–44)
	(ii) Familiar food: expensive and hard to find	‘when it comes to veggie, we have some that we…we find them in African stores but if we want to live such a lifestyle you need to spend a lot as well. Yeah, importation is, you know… the currency rate as well.’ Participant 04FG (focus group participant, man, age range 35–44)
The overwhelming in-store supermarket experience	(i) Cognitive overwhelm and unintended purchases	‘I always feel really overwhelmed…it’s loud, it’s noisy, erm, you know, there’s lots of people…there’s not enough space I guess’ (Participant 35, woman, age range 35–44). ‘I suppose it’s quite, it’s quite hard work because, because the prices have kind of increased so much and, yeah, kind of spending more time, I think, having to kind of think a bit more and, and kind of, you know, actually browsing more than I probably used to.’ Participant 44 (man, age range 35–44)
	(ii) Reducing temptation through online shopping	‘with the online shopping I feel like I’ve got a bit more control in that em, I have not got a number of different colorful things to distract me and you know, I think… I can be more prescriptive in what I choose when I’m online and be careful about what I choose, I tend not to get distracted, you know, you are not walking off into other aisles’ Participant 11 (woman, age range 35–44)

**Table 3:** Main theme, sub-theme, and example quotations, an unanticipated consequence of budget maximizing shopping practices.

Theme	Sub-theme	Example quotation
Unconscious, environmentally sustainable shopping practices	(i) Food is too precious to waste	‘if I bought like a whole watermelon, erm, what’s the chance I’m going to manage to finish it before it sort of goes out, goes bad and stuff…[I buy] mainly just the longer shelf life things. So more tinned stuff, you know, more frozen stuff, stuff that lasts a lot longer than, you know, fresh stuff, basically. And that’s just literally due to the fact that you cannot just afford to throw stuff away’ Participant 29 (man, age range 25–34)
	(ii) Meat is a treat	‘A lot of the time the food is too expensive and as I’ve said about fresh meat and stuff as well costing so much, eh, money that it, it, I will find alternatives or else go vegetarian for a few meals a week in order to cut the costs down.’ Participant 21 (woman, age range 35–44)
	(iii) Shopping cheaply means walking	‘I’ve got the time, you know, to shop around cheaply and do the best I can and to travel to Lidl, which is only 25 min walk. But then I do get the bus back.’ Participant 08 (woman, age range 45–54)

Our sixth theme, with three related sub-themes, was revealed as an unexpected consequence resulting from discussions surrounding shopping practices participants enacted, in order to help maximize their food budget when trying to buy healthy food in the supermarket. This theme was not consciously described by any participant; rather, it is an observation of the research team and therefore, is presented separately (Table 3).

### 3.3 Theme 1: deals

All participants spoke about supermarket deals during the interviews and focus groups. Some participants described deals positively, as providing the opportunity to purchase food items that would otherwise be unaffordable given their limited budget. However, deals were also perceived negatively by others who described how they hindered their ability to follow a healthy eating plan, because the offers changed frequently. The healthiness of the food typically associated with supermarket deals was also questioned.

#### 3.3.1 The good

Participants talked about the beneficial aspects of supermarket deals and offers in reducing the price of food items. For example, Participant 05, who described ‘hopping around’ various supermarkets to maximize his limited food budget, detailed how deals and offers within these different stores helped provide the opportunity to try (often otherwise unaffordable) new or healthier foods:

‘*sometimes it'll be something where like maybe I've thought about buying it before but I've never wanted to because I wasn't like too sure about like the prices of it or it was just too expensive in the past and now it's a bit cheaper. So I'm like ohh, OK, I'll, I'll definitely look into that*.’

Participant 05 (man, age range 18–24)

Participants often spoke about the usefulness of healthy deals and how discounted supermarket offerings, commonly ‘*wonky fruit and veg boxes*’, available from various stores, allowed them to afford to purchase the fresh fruit and vegetables they wanted to include within their diet. During her interview, Participant 54 expressed a desire to continue making healthy meals from scratch, as she had done for many years, and reflected on the use of deals that allowed her to continue to achieve this:

‘*the things that I particularly like are, in Morrisons you can get a wonky veg box. I don't care what the carrots and leeks and whatever, I really don't care what they look like …One that I got recently, was the wonky sweet potatoes. Fantastic, I got something like eight sweet potatoes for under a pound, and that's great, I can make soup, I can make wedges, I can make mash, I can do, put them in casseroles. I don't care what they look like*.’

Participant 54 (woman, age range 45–54).

#### 3.3.2 The bad

Participants described healthy food items as often only being available at a reduced price for a limited period and conveyed frustration around the fluctuating nature of supermarket deals. Participant 02 shared how her initial enthusiasm around purchasing and incorporating a healthy food item (affordable while on offer) into her weekly shop dissipated once the offer finished and she realized she could no longer afford it, leaving her with a sense of futility. She went on to share that this meant she was left living off highly processed, cheap foods, which she knew were harmful for her health.

‘*I try, but it doesn't really get me anywhere. Because, you know, I might do it one week when something is in, something that is healthier, is on offer and then that'll be for four weeks. And then by the time that is not on offer anymore, there’s no way…I could do it for four weeks, four weeks isn't gonna’ have, is no, it’s that drop in the ocean you know, so it's just pointless. So in the end it's like it's like it's just close your eyes, buy what you can afford and carry on.*’

Participant 02 (woman, age range 45–54).

Participants also described how products on offer, such as the wonky fruit and vegetable boxes, regularly sold out or were not routinely available. This occurrence appeared to hinder participants from purchasing fresh fruit and vegetable items at an affordable price point:

‘*the Lidl that I, is local to me, they used to do a box of veg for £1.50, but they've stopped doing it now, it was really hit and miss when they were doing it’*

Participant 08 (woman, age range 45–54).

Furthermore, participants perceived supermarket deals and offers as often not available on items they routinely purchased. Participant 17, a single parent with two young children, shared her frustration that discounted prices on fresh fruit and vegetables tended to be on less familiar items that her children may not eat, rather than items with which she was more familiar:

‘*Lidl and Aldi do have like the super six thing that they do every week but, I find the stuff on there's just not stuff like you'd eat in every day sort of situations. It'll be like, I don't know, like asparagus or something, it's like, well, what I do with that? I've never ate it before. I don't think my kids would eat it. It's very rare that you'd get like your basic, like, you know, like apples, carrots, that kind of thing, it's, it's always more the different, erm, veg*.’

Participant 17 (woman, age range 18–24).

The majority of participants described purchasing ‘yellow sticker’ or reduced food items, often close to their expiry date. However, perceptions of potential risks to health from purchasing and consuming such items meant some participants did not engage with these types of offers. For example, Participant 57 described how rising food prices and financial constraints negatively impacted his ability to buy the healthy, nutritious food he and his family required for ‘healthy living’. He shared his reluctance to buy food items reaching the end of their shelf life, given the potential for such foods to be bad for his health:

‘*I can't be going for a two for one offer for some fruit that I know is going to get spoiled...within a couple of days because the price is cheap…that would be detrimental to my health. It's like buying sickness with your money.’*

(Participant 57, man, age range 35–44).

Similarly, Participant 08 used a personal anecdote to describe why they were put off buying reduced-price food in the supermarket due to concerns around the potential for such food items to make them physically unwell:

‘*I do buy food that's reduced, but I've gone off doing that because I had food poisoning from some fish that I got from the, you know, local shop Co-op’*

Participant 08 (woman, age range 45–54).

#### 3.3.3 The ugly

There was a general consensus among participants that most of the cost-saving deals and offers, involving larger price reductions, were associated with less healthy, ‘junk food’ items compared to healthier products, including fruit, vegetables, and seeds. During their interviews, participants demonstrated a good understanding of the components of a healthy diet, yet many explained it was difficult to avoid purchasing cheap deals on less healthy foods as these appeared to be more prominent within the supermarket space, as illustrated in this quote:

‘*I very rarely see deals when it comes to fruit and veg. Erm, I think sometimes you can get a deal where you do like the stir fry thing where you get your meat and you get the noodles and things like that, so I guess that would be like a multi-buy thing, but I don't really see it on anything else, it's always like your Cadbury's, or Coke-a-Cola or, you know, buy one get one free on pizzas, you know, the frozen pizzas or whatever it might be, so yeah, I definitely think that they target that, it's not here, have some nutritious flax seed, you know…It's never the staples, is it, it's always the junk they're trying to sell you*.’

Participant 35 (woman, age range 35–44).

However, at the same time, participants believed that their perception of increased supermarket deals and promotions on less healthy food items could be due to their personal taste preferences and purchasing habits. As highlighted in the following quote by Participant 05, there was a sense that an individual’s attention might naturally be drawn to deals on foods they typically purchase or whose taste they prefer (i.e., generally less healthy food items):

‘*I think the ones I'd recognize would be on more unhealthier foods, probably just because that's the food I like, and maybe there are healthier food eh, savings promotions that are on, that maybe I just don't see or I just kind of ignore because I'm, I’m not really gonna get that… there could be er, more options. I feel like maybe, er, a lot of the time if I am, er, if I do look around, I don't see healthy foods as much but, I'm not just, I'm not sure if that's just ‘cause my habits mean that, er, I kind of pass those sections or if it's actually ‘cause the, maybe er, maybe there, just there's less of that stuff available*.’

Participant 05 (man, age range 18–24).

### 3.4 Theme 2: skepticism about supermarkets and wider food system

While Theme 1 reflects participants’ observations around a specific element of the supermarket experience (deals and offers), Theme 2 focuses more broadly on the supermarket as a whole and its situatedness within the wider food system. Participants expressed varying opinions on the role of supermarkets in keeping food prices affordable, especially for those on a low income. While some suggested supermarkets prioritize profit over public health, others described the supermarket as a business, with the necessary goal of making a profit. The situatedness of supermarkets within the wider food system, where price reductions in this one area could have (negative) impacts elsewhere, was also recognized. Overwhelmingly, when asked, participants identified upstream, Government-level change as the critical lever to help ensure the affordability of healthy, environmentally sustainable food items for all members of society.

#### 3.4.1 Skepticism in the aisles: disingenuous discounts and deals

During the interviews and focus groups, participants expressed skepticism about the fairness of food prices, often viewed as solely determined by the supermarket. Such beliefs led the majority of participants to view supermarket deals and offers as disingenuous, stating that supermarkets could remain profitable even if food prices were lower. Despite declaring her loyalty to shopping with a large UK supermarket chain, having previously been employed by them, Participant 02 expressed distrust in the supermarket’s food pricing strategies:

‘*I think, because of this cost of living crisis, they’re getting away with not passing any savings on to consumers because they’re just going well, it’s costing us more to bring in, you know, it might have gone down by, I don’t know, 20p per head of lettuce that week, they don’t reduce, reduce it by 10p to help us, they will keep it the same level or even increase it because they know they’re gonna sell, which means they make more profit.*’

Participant 02 (woman, age range 45–54).

However, the sense of skepticism did not always apply equally to all pricing strategies. For example, on one hand, Participant 44 perceived price promotions, such as buy-one-get-one-free multibuy offers, as genuinely allowing him to acquire a larger amount of food at a good price:

‘*if it's money off, at the end of the day, I'm still taking home the same size or the same product, you know, but I think when it's a multi-buy, you know, you kind of feel you've got more in your trolley*’

Participant 44 (man, age range 35–44).

In contrast, this same participant expressed a sense of distrust around the pricing of food reduced under supermarket loyalty card schemes:

‘*you know like the Clubcard, with Tesco they do the Clubcard prices… Asda Rewards they kind of do these star products and things but you never feel that, you always feel like you are just paying the original price anyway and they have just made it look cheaper.*’

The interview data suggested a strong sense that participants viewed supermarkets as favoring profits ahead of the health of their customers. Participant 02 talked to, what she believed to be, the injustice of supermarkets making huge profits. Calling for a shift in perspective from retailers, Participant 02 asked them to consider that consumers are ‘*not just profits, there are actually people on the end of these pounds that are coming in’*. She continued:

‘*for some of us it is the, the few pounds that we have got, you know, so we need to, they need to look after the little people, not just the people with, that are lucky enough to still have the funds because we’re not all them. They don’t ever seem to think about the little people, and that’s what really annoys me. Because, you know, we, we’re as much, we are paying as much as everybody else but we haven’t got that money to use.*’

Participant 02 (woman, age range 45–54).

Participant 35 expressed her frustration at the ableist perspective she believed was held by many individuals in positions of power. She conveyed a sense of anger toward supermarket bosses and those in Government who, she perceived, suggested that PLWO and FI just needed to concentrate, try harder, and stop being lazy when it came to food purchasing and preparation. Participant 35 argued that those in power who had the potential to provide support and alleviate some of the struggles faced by those living on a low income related to purchasing and consuming a healthy diet lacked the lived experience, understanding, or empathy to work toward finding solutions:

‘*I think the people that own these companies, like Tesco, and the people that are in the government, are a certain type of people, who had a certain type of upbringing, and they don’t understand what it’s like to live in social housing, with hardly any money, with a disability or some other restrictive factor and I think that that is hugely problematic because, you know, if you’re tired and you’re disabled and you physically can’t do anything, you’re always going to chuck in a pizza rather than make, you know, a fancy dish.*’

Participant 35 (woman, age range 35–44).

#### 3.4.2 Supermarkets are only part of the problem

While expressing skepticism over whether supermarkets were doing all they could to keep prices low for consumers, participants also often acknowledged that supermarkets sit within the wider food system, where multiple factors interact to determine the price of goods. During her interview, Participant 12 spoke about the rising cost of food, fuel, rent, and other household bills as an economic consequence of COVID-19 and the war in Ukraine. She considered the potential impact of the wider economic climate on agents within the food system that might play a role in determining food prices, acknowledging that supermarkets may not always be in control:

‘*it is not always on the supermarkets, you know, it is the state of the whole, you know, cause obviously, if there is like this energy crisis the farmer has to pay much more for the electricity, for their water, you know’*

Participant 12 (woman, age range 45–54).

Others expressed concerns about potential unintended consequences of demanding cheaper food, suggesting that efforts to drive down food prices could result in unethical practices within the food system. Participant 11, who had recently experienced a change in her financial circumstances following the breakdown of her marriage, described how, before budgetary constraints restricted her purchasing behaviors, she had previously prioritized environmental sustainability when shopping in the supermarket. She voiced uncertainty around the feasibility of reducing food prices without compromising another part of the wider food system:

‘*I think where meat and fish are concerned, I think it’s really, really hard for them to bring the prices down because then other people aren’t getting paid and well, you know, welfare gets cut so I, I, I don’t, I don’t know what, I don’t know what can be done about, about that*.’

Participant 11 (woman, age range 35–44).

Furthermore, Participant 08 questioned whether we should expect supermarkets to change at all regarding helping support the purchase of healthy, environmentally sustainable food items for PLWO and FI, given that the primary role of their business is to generate profit, as opposed to Government assistance programs or charitable organizations:

‘*how are you gonna get supermarkets to assist because frankly they’re not in, they’re not social services, are they?...they’re not, yeah, they’re profit and food distributors and they’re not kind’*

Participant 08 (woman, age range 45–54).

#### 3.4.3 Upstream change is required

When asked what they thought could help support PLWO and FI access healthy, environmentally sustainable food from the supermarket, almost all participants stated that upstream, Government-level change was required to alleviate the burden of high food prices. Discussing this issue during one of the focus groups at the foodbank, one participant spoke of the need for the Government to consider the impact of fiscal policy on all citizens:

‘*the poor are really finding it hard to be able to make ends meet so, yeah, the government have a role to play, maybe bulk of the role is on the government...if they can be sensitive to the needs of everyone around on the streets to, to work on the exchange rate so that at least, and to work on the inflation rate, so that everyone at least can be comfortable’*

Participant 05FG (focus group participant, man, age range 35–44).

Another participant, who reported challenges around consistently being able to afford and consume a healthy diet, called for support from the Government to enable all members of society, including those living on a low income, to follow healthy eating recommendations. She stated that governments ‘*are always telling us that what we should eat’* and argued they should not make healthy eating recommendations without providing citizens the support necessary to realize these, emphasizing ‘*they have gotta do something’*. This participant stressed their belief that any Government intervention would need to be tightly regulated, otherwise supermarkets would find loopholes allowing them to continue raising the price of food:

‘*They’ve [government] got to do something about it because the supermarkets will find a way out, they’ve got lawyers that will find little tiny loopholes in, even though in, erm, you know, the, the stuff that comes out of the government, they can find ways around it, but hopefully then they’ll be penalized. But you know for, to make sure, hopefully that it happens and it happens properly, it needs to be done from on high’*Participant 02 (woman, age range 45–54).

Other suggestions around ways in which upstream change might be enacted included controls or a cap set by the Government on the price supermarkets can charge for items. One participant, a non-UK national, stated that a system similar to their home country should be enacted:

‘…*they should have a price control. In my country, they have a price control. You can’t just, say for example, this is how the, the price is…Lidl should have, Marks and Spencer should have, everybody have a price across, they will not differ from the other*’

Participant 06FG (focus group participant, woman, age range 65+).

To help with the purchase of a healthy diet while in receipt of Government assistance, such as Universal Credit (UC) in the UK, one participant, a UC recipient who described heavy restrictions around the healthy food items she was able to afford to buy, argued Government should extend the support offered to families with young children (i.e., vouchers for fresh fruit and vegetables) to individual claimants. However, the following quote from this participant talks to the humiliation and the stigma she sees as attached with utilizing such a scheme, perhaps reflecting the desperation of some living on a low income to acquire fresh fruit and vegetables:

‘*I know that the Alexandra Rose charity supports families with vouchers, so you can get fruit and veg from street markets, um, in London if you’ve got kids, but I would like to see some support for single people on Universal Credit without a family because I feel like, I get no support at all…It…It’s humiliating, giving people vouchers. I don’t wanna have a voucher, but I don’t think the government would agree to up Universal Credit so that people could buy more fruit and veg*.’

Participant 08 (woman, age range 45–54).

### 3.5 Theme 3: other people’s role in enhancing or undermining healthy diet intentions

The impact of others, usually another member of the household or a close family member, on the shopping experience was highlighted. Participants shared both positive impacts, such as guiding some toward healthier items, and negative impacts, such as feeling compelled to purchase less healthy food items in line with the food preferences of other members of their household (children, partner/spouse), which did not support the participants’ weight loss intentions and goals.

#### 3.5.1 Steered toward healthy purchases

Participants described how another family member, in the following quotes, a sister and a partner, helped steer them away from typical, less healthy purchases toward healthier food items. Participant 10, who lived with and shopped for his dad and sister, described the positive impact of his sister’s presence when she accompanied him on his trips to the supermarket, compared to the decision-making and purchasing patterns undertaken when he shopped alone:

‘*when I go by myself I’m much more likely to go toward like the frozen food section or like to buy like prepackaged food which I can just, you know, put in the oven or microwave and just make, but when she [sister] comes along she tends, we tend to have more of an effort to go toward like the fresher food aisle. So, for example, like chicken breast in Tesco, they have like a halal section so we tend to go there every time when, when she comes along so we can buy stuff from there and that probably does have an effect like I’m less likely to then go to the frozen food sections because we're buying these foods instead whenever I go with her. So, yeah, no, I would say there’s a difference in decision making for sure, to an extent*.’

Participant 10 (man, age range 18–24).

This second quote describes the impact of a partner on the participant’s food purchases. While ‘nannying’ or being steered toward making healthier purchases may not always be welcomed, the benefit of this influence, in terms of supporting healthier food choices, was acknowledged:

‘*my partner is a lot slimmer than me and doesn’t kind of have like the weight issues that I do, and I think he always kind of tries to steer me away from some bad things…if there’s kind of promotions and stuff like that, on stuff that’s not particularly good for me, I probably sort of tend to stock up on it, whereas he wouldn’t…on one hand, you know, like everybody else, I don’t really like being told what to do but, at the same time, it kind of, I suppose it does reign me in a bit as well*.’Participant 44 (man, age range 35–44).

#### 3.5.2 Decreased healthfulness and sacrificing healthy eating aspirations

In contrast, some participants spoke about the negative impact others had on their purchasing decisions. There was often a sense that participants with children sacrificed their own healthy eating aspirations and purchased less healthy foods and snacks that aligned with their child’s taste preferences to reduce the potential for food waste. Participants viewed the prioritization of others’ food preferences and the inability to simultaneously purchase healthy food items they wanted to eat as a barrier to their weight reduction intentions:

‘*they’re [teenage children] quite fussy eaters, to tell you the truth. So, a lot of the time I’m getting stuff, like they like frozen pizzas like and it’s not helping with me weight and stuff like that*.’

Participant 46 (man, age range 45–54).

This sense of self-sacrifice was not only described in parent–child relationships but also within partner or spouse relationships. Participant 45 spoke of the challenge of shopping healthily on a low income, given their partner’s appetite and the requirement to buy less healthy snacks to keep him satiated. This participant expressed a desire to purchase the ingredients required to make healthier versions of snack food items, but described being unable to afford to do so, resulting in the purchase of the cheap, unhealthy versions of these products available in stores and within their budget:

‘*we do try to not get processed stuff but, like particularly my husband, he’s just always hungry...to try and sort of fill him up and stuff, like, I do buy quite a lot of kind of freezer foods that do tend to be processed…He’ll be picking up…basically picks up all the cheap stuff that’s on offer that’s really bad for you but will fill him up… I try not to pick up Supernoodles and like the pizzas and the processed stuff, but then he complains that there’s nothing to eat, and there’s no snacks when he’s hungry before dinner’*

Participant 45 (woman, age range 35–44).

### 3.6 Theme 4: financial restrictions facing non-UK nationals

The food bank from which focus group participants were recruited, based in an urban location in North East Scotland, was predominantly attended by non-UK nationals. The majority of the non-UK nationals who participated in discussions had come to the area to study or work. Discussions with this group reflected additional hindrances to purchasing a healthy, environmentally sustainable diet, such as a lack of access or no recourse to public funds (UK Government financial assistance such as Universal Credit, Tax Credits, or Child Benefit), and the high costs associated with accessing familiar, healthy food items from their home country.

#### 3.6.1 No recourse to public funds

Participant 57, who had come to the UK on a work visa with his wife and children, spoke of his frustration around the specific challenges faced by those who were not entitled to Government financial assistance, despite paying taxes and money toward the health service:

‘*let me tell you one of the struggles of people of our own descent face here, most of our visa carries an exemption, and what is that exemption, not entitled to public funds, and that places a huge financial burden on us immigrants specifically. Now, every other person that is a citizen, or has whatever status, can benefit from all those things, all those government interventions, but we can’t because we are not entitled to public funds. And why we are not, I cannot see because, I’ll use myself as an example, I pay my tax, right, I live in the country legally, I was employed by an organization with a five year visa sponsorship, right. I pay for NHS, I mean, this is about £3,000, over £3,000 that we paid for NH, I mean for a period of that five years’.*

Participant 57 (man, age range 35–44).

#### 3.6.2 Familiar food: expensive and hard to find

Healthy, environmentally sustainable food was described as expensive by non-UK nationals. However, familiar food from their home country, such as certain vegetables, was described as being even less affordable due to importation costs:

‘*when it comes to veggie, we have some that we…we find them in African stores but if we want to live such a lifestyle you need to spend a lot as well. Yeah, importation is, you know… the currency rate as well*.’

Participant 04FG (focus group participant, man, age range 35–44).

Furthermore, familiar foods were described as difficult to source, adding yet another barrier to purchasing the healthy food items they would like to buy:

‘*I’m African, from Sudan, then there is some item that we needed, I used to go, when I used to go back home for holiday, I can bring some items. However, now I couldn’t cause of there is a war there, so then I need, if I need, if I really need to have that part then I need to shop it from Facebook or some people who, you know, because there is no, I will not find it here in Aberdeen so I need to go to Glasgow or to ask it from the Facebook*’

Participant 55 (woman, age range 35–44).

One parent described making efforts to prioritize their children’s needs and preferences for familiar foods at the expense of their own to keep food costs affordable:

‘*being an African, you know, most of our meals are not really in the stores. So, we get to pay high prices to actually get our own meal. Yeah, so it’s really, and then, you know, the children, for me, I have kids…I’m really bent on going to get what we, they are used to and then we gradually make adjustments for our own meal*.’

Participant 05FG (focus group participant, man, age range 35–44).

### 3.7 Theme 5: the overwhelming in-store supermarket experience

Participants identified several aspects of the supermarket environment itself that could potentially hinder the purchase of healthy, environmentally sustainable food items. Participants often described feeling a sense of cognitive overwhelm when shopping in-store, further compounded by the requirement for constant mental effort expended through calculating the best prices to maximize their limited budget. Enticing deals on less healthy food items, marketed in ways that made them hard to resist, were said to increase the likelihood of purchases that participants had not planned to make. Participants described various coping strategies employed to try to manage the overwhelming environment and prevent unintended purchases.

#### 3.7.1 Cognitive overwhelm and unintended purchases

Discount supermarkets, which were used by many participants (e.g., Aldi and Lidl), were commonly described as overwhelming, ‘*I always feel really overwhelmed…it’s loud, it’s noisy, erm, you know, there’s lots of people…there’s not enough space I guess’* (Participant 35, woman, age range 35–44). This sense of cognitive overwhelm was echoed by another participant who talked to the additional struggles that she (as someone without children) perceived were being faced by those navigating the crowded supermarket space with their children in tow:

‘*In Lidl’s it can just feel like there’s people kind of, you know, you’re fighting through a sea of people, it’s quite busy, and I think people in like, I don’t know, but whenever we go to Lidl’s it’s these people with kind of like, it’s just people are strug, like they tend to have kids with them, I think they’re quite busy people and, there’s a lot going on and they’re maybe not so aware of what’s around them cause they’re trying to do too many things at once*.’

Participant 45 (woman, age range 35–44).

Amid this overwhelming environment, participants living on a low income also described having to try to make sense of often confusing pricing information. This speaks to additional work and cognitive burden placed on those who are required to spend time examining food prices to ensure they are fully maximizing their budget.

‘*some of these deals are confusing, you have to look at price per weight rather than price per unit to, you know, properly compare’*

Participant 36 (woman, age range 55–64).

Participant 44 talked about the impact this additional work had on the time it took them to complete the shopping trip, suggesting the increased cognitive burden experienced in the supermarket led to an increased physical burden:

‘*I suppose it’s quite, it’s quite hard work because, because the prices have kind of increased so much and, yeah, kind of spending more time, I think, having to kind of think a bit more and, and kind of, you know, actually browsing more than I probably used to*.’

Participant 44 (man, age range 35–44).

Participants also often talked about feeling lured into making unintended purchases, most commonly on less healthy food items. Participant 11 described being ‘caught up’ in the various deals and offers available when shopping in-store. She reflected on the inner turmoil she experienced in the store whilst trying to remain disciplined and purchase only what she intended and not make any impulse buys:

‘*the in shop experience is, um, I feel like I’m fighting myself a lot of the time, which is a bit, a bit weird, um, so yeah, I’m not, I’m not great with in store shopping unless I’ve kind of, um, had a word with myself before I go in… I mean, I’m talking about cupcakes or something now, how can you get caught up in the moment over cupcakes. But you know what I mean? It’s erm, I try and be really, really disciplined with shopping nowadays and I do find that, I do find that times I buy stuff that I didn’t intend to and I, I feel like I’ve been tricked, if you know what I mean?*’

Participant 11 (woman, age range 35–44).

Distraction techniques or coping strategies aimed at maintaining focus to support the purchase of planned food items and avoid impulse buys, such as the use of music, were described. However, Participant 11 recounted how such strategies are not always effective. Factors such as tiredness have the potential to allow supermarket promotions and marketing to entice the unplanned purchase of less healthy food items:

‘*I get very, I get kind of I don’t, it’s like a sensory overloading, I don’t really know what happens. I’ve started to try and listen to music when I’m in supermarkets to try and keep me kind of focused in one spot and just concentrate on what I’m, I’m, you know, I need to do, particularly if I’m tired, um, which is quite a lot. I find that I, I lose track. I start wandering and then yeah, you think, oh, what’s this new kind of jelly? And I had no interest in jelly at all but it looks nice so, you know, and I do tend to notice new products*.’

#### 3.7.2 Reducing temptation through online shopping

Another coping strategy utilized to avoid temptation was shopping online. Two participants, both regular online supermarket shoppers, expressed a sense of being in greater control of their purchasing behavior when shopping for food online. They stated the online environment better enabled them to purchase only those food items they intended to buy due to fewer tempting distractions within this space, compared to those typically encountered within the physical supermarket:

‘*with the online shopping I feel like I’ve got a bit more control in that em, I haven’t got a number of different colorful things to distract me and you know, I think… I can be more prescriptive in what I choose when I’m online and be careful about what I choose, I tend not to get distracted, you know, you’re not walking off into other aisles*’

Participant 11 (woman, age range 35–44).

Participant 33 talked about the ability to use filters when shopping online, further reducing exposure to tempting, less healthy foods, which are unavoidable in the physical supermarket space:

‘*and I tend to, for some reason, I’m much more controlled with both my spending, also the food I tend to buy. I do tend to be healthier if I’m shopping online. I don’t know why that is, and maybe it’s just not the temptation’s not like right in my face, I don’t know… If I’m in store and there’s, you know, an offer on, cause I’m vegan myself, if, if there’s an offer on, I don’t know, like a vegan cake or something, I’m just terrible. It’s really, really bad, whereas I guess, because I use the filter, I don’t see them online. I hide it so it’s not, they’re hidden away, so I don’t know if they’re on offer*.’

Participant 33 (woman, age range 25–34)

### 3.8 Theme 6: unconscious, environmentally sustainable shopping practices

One final theme we observed in the data, *unconscious, environmentally sustainable shopping practices* was identified as an unintended consequence of the strategies participants used to help maximize their limited food budget. When asked about environmentally sustainable shopping practices, participants described a desire to engage through purchasing products with recyclable packaging, locally grown fruit and vegetables, and plant-based meat alternatives, but expressed (often regretfully) an inability to do so due to the high cost of such food items. However, participants’ budget-maximizing shopping behaviors often reflected environmentally sustainable shopping practices and behaviors, including the potential for reduced food waste, consuming less meat, and using environmentally friendly modes of travel (i.e., walking and public transport) when getting to and from the store.

#### 3.8.1 Food is too precious to waste

A commonality of discussions with participants was the sense that food was too precious to waste. Shopping online, for some, was seen as a gamble; there was the chance for substitutions for products that may not get consumed, or for products too close to their use-by date that would end up being thrown away. Participant 17, a single parent of two young children who worked to maximize her food budget by buying food items she was certain would not expire before they had the chance to be consumed, described a move back to in-person shopping due to her frustration at the short shelf life of fresh produce ordered online:

‘*I used to shop online. Not so much anymore. I found that things would get substituted and it wouldn’t be quite what I wanted and, obviously, the dates and stuff as well, when you order online, you tend to get short dated, which would annoy me cause I tend to like shop and then like the first big shop I do has to obviously last longer than the little top-up shops and it just wasn’t lasting cause the dates were just too short*.’

Participant 17 (woman, age range 18–24).

Participants described buying longer-lasting, tinned, and frozen produce or limiting the quantity of fresh fruit and vegetables. The following participant described the rationale for such purchasing patterns as being driven by concerns around food waste, given he lived alone and therefore was unable to consume fresh fruit or vegetables in their entirety before they expired:

‘*if I bought like a whole watermelon, erm, what’s the chance I’m going to manage to finish it before it sort of goes out, goes bad and stuff*…[I buy] *mainly just the longer shelf life things. So more tinned stuff, you know, more frozen stuff, stuff that lasts a lot longer than, you know, fresh stuff, basically. And that’s just literally due to the fact that you can’t just afford to throw stuff away*’

Participant 29 (man, age range 25–34).

#### 3.8.2 Meat is a treat

Meat was often viewed as a treat or a luxury that many could no longer afford, despite participants recognizing its important nutritional qualities, ‘*I know meat are important for our proteins, you know, but because it’s, the price is quite high we just cannot, we just cannot afford it’*, Participant 06FG (focus group participant, woman, age range 65+). Participants also often contrasted their inability to buy meat, despite its availability in stores, with prior periods of their life when such food items were more easily affordable and a regular part of their diet. During their interview, Participant 10 reflected on having been very health-conscious before COVID-19; however, since then, he described challenges around his ability to afford to return to eating the healthy foods he once regularly consumed:

‘*if I think back to when was the last time I actually made the grilled fish myself, like two months ago, compared to when I used to have it like two, three times a week for like years on end. So, they have the food, I just, I just don’t buy them as often and I don’t eat them as often’*

Participant 10 (man, age range 25–34).

The reduction in their purchase of meat products led some to reflect on major shifts in their dietary patterns toward a more vegetarian-based diet:

‘*A lot of the time the food is too expensive and as I’ve said about fresh meat and stuff as well costing so much, eh, money that it, it, I will find alternatives or else go vegetarian for a few meals a week in order to cut the costs down*.’

Participant 21 (woman, age range 35–44).

#### 3.8.3 Shopping cheaply means walking

Participants described walking to the supermarket as a way of saving money in order to maximize their food budget. Where necessary, public transport or a taxi, whose costs were factored in to ensure the use of this transport was affordable, was used for the return journey. Participant 08 reflected on the time consuming nature of such cost saving behavior, a consequence with which she felt she was able to contend, perhaps due to her status as a single person without any dependents and currently unemployed:

‘*I’ve got the time, you know, to shop around cheaply and do the best I can and to travel to Lidl, which is only 25 minute walk. But then I do get the bus back*.’

Participant 08 (woman, age range 45–54)

The time-consuming and effortful nature of saving money by walking to the store is further exemplified in the following quote, which also demonstrates the careful planning undertaken to ensure any transportation costs do not detract from the overall savings incurred from doing their ‘big shop’ at one particular store:

‘*I’m heading to this store, hour and a half…we’ll walk, walk there. We’ll go around, we’ll do the shop and we’ll make sure it’s a big shop cause we need to get these things. But we can’t go there all the time and getting home is gonna be a pain anyway so we’ll try and make sure that we’ll get, like, an Uber home or something like that… So that’s gonna be 8-9 quid to get home for that, so we have to make sure that the savings add up to that’*

Participant 18 (man, age range 45–54).

## 4 Discussion

The aim of this study was to better understand what helps and/or hinders PLWO and FI, to purchase healthier, environmentally sustainable food from the supermarket to meet their personal weight loss or weight maintenance goals. Discussions revealed PLWO and FI are forced to make difficult choices when shopping, and how factors that help one person (i.e., supermarket deals, other family members) may hinder the purchase of healthy food for another. While participants predominantly expressed skepticism toward supermarket pricing strategies, they acknowledged the impact of the wider food system on food prices, including rising costs associated with producing food, supporting farmers’ livelihoods, and ensuring animal welfare. Discussions also revealed that budget maximizing strategies, although potentially resulting in more environmentally sustainable food purchasing behaviors, are not enacted through choice and do not necessarily align with participants’ shopping aspirations to purchase a healthy diet.

Participants described supermarket deals as both helping and hindering the purchase of healthy food in the supermarket. For some, deals were described positively, ‘*the good’*, providing participants the opportunity to buy healthy, nutritious food or to try new foods they otherwise could not afford. Deals, therefore, may provide nutritional benefits and add variety to what might otherwise be a very restricted diet. However, the fluctuating nature of deals, where a product is on offer and affordable 1 week but not the next, ‘*the bad*’, was highlighted as a hindrance to adopting and maintaining healthy purchasing and consumption patterns, a requirement for weight loss and health and weight maintenance (49, 50). Additionally, participants described deals as more frequently available on produce that they did not typically buy and consume. People living on a low income may not have the budget to buy unfamiliar produce that does not match their or their families’ food preferences and risk going uneaten (51, 52). This may inhibit their desire to engage with certain supermarket deals. There was also the notion of ‘*the ugly*’ side to supermarket deals and promotions, including participants’ perceptions of an increased number of deals or greater reductions on less healthy compared to more healthy food items. While participants often reflected that this perception may have been shaped by personal food tastes and preferences, creating a bias leading them to notice deals and offers on less healthy products, research conducted in stores supports participants’ initial concerns. The Food Foundation Industry report found that 41% of price promotions and 27% of multibuy deals in supermarkets were on high fat, salt, sugar (HFSS) foods, compared to only 3% of price promotions and 4% of multibuys on fruit and vegetables (53).

In line with existing research involving PLWO and FI (54), participants in the current study commonly spoke of their distrust of supermarket pricing strategies and the notion of disingenuous deals and promotions, which were perceived to maximize supermarket profits rather than support consumers to maximize their budget and acquire food at a fair price. A review of supermarket loyalty card prices, conducted by the Consumer Marketing Authority (CMA), found 55% of consumers surveyed believed reduced price products, available only to loyalty card holders, reflected inflated prices for non-members rather than genuine savings (55). While the review found 92% of loyalty deals could result in average savings of 17–25% for consumers, it also revealed loyalty prices were not always the cheapest and, to fully maximize food budgets, consumers are required to shop around (55). People living on a low income commonly shop where they can, rather than where they would prefer, and acquiring the food they want to buy at a price that fits their budget often involves visits to multiple supermarkets (21). *Skepticism about supermarkets* and supermarket deals possibly stems from their wide knowledge of food prices across the different stores. On the one hand, such knowledge may help them maximize their budget and acquire healthy food at the best possible price, on the other hand, the requirement to shop in multiple stores leads to time consuming, effortful shopping practices. The shopping practices of PLWO and FI are enacted in the face of restrictions and sacrifices, all of which can take an emotional toll and have negative consequences for the individual’s mental wellbeing (21). Perhaps unsurprisingly, while food and grocery businesses were once one of the most trusted sectors among consumers, trust in this industry has witnessed a decline since 2021 (56). Although the majority of participants expressed a belief that the supermarket should be doing more to help customers living on a low income, some spoke to other agents within the wider food system that played a role in determining the price of food in the store.

*Acknowledging the wider food system*, some participants expressed uncertainty around how much control the supermarket had in setting the price of food, given the rising cost of commodities such as energy, something which has impacted the price of food production, storage, and transportation (57). In the UK, public funds for farming are declining, and profits are marginal (58). For every £1 spent in store, farmers often make less than 0.01p for the food they produce (58). Participants recognized the need to pay farmers fairly for their produce and the need for fair prices that ensure the welfare of livestock. It was also acknowledged that supermarkets are businesses, designed to make a profit, and do not exist as part of Government social services. The majority of participants suggested upstream, Government-level policy intervention, such as the introduction of Universal Basic Income or increased welfare provisions, as the most ideal pathway to helping support PLWO and FI purchase healthy, sustainable food. However, this intervention suggestion is limited as non-UK national households with no recourse to public funds face increased financial pressures and challenges in acquiring healthy, environmentally sustainable foods. There have been longstanding calls from campaigners for this policy to be abolished, arguing that the legislation deprives those in extreme poverty of vital support, disregards people’s dignity as human beings, and reinforces social exclusion of migrant communities (59).

Prioritizing the food preferences or dietary requirements of other household members was commonly discussed during the interviews and focus groups. The influence of other members of the household was described as helpful in promoting healthier dietary choices for some, whilst hindering such purchases for others. The positive influence of other family members on food purchasing decisions, in terms of *enhancing* healthier food choices, steering the participant away from less healthy in-store promotions or food purchases that did not align with their weight loss or weight maintenance goals was described. In contrast, others reflected how forgoing their own food preferences and requirements to meet or prioritize the needs and preferences of other members of the household, *undermined* their attempts to eat a healthy diet in order to reduce or maintain their own body weight. These findings align with existing research, which suggests parents living on a low income often prioritize family food preferences and cost ahead of product healthfulness, purchasing familiar, high-energy-dense, less nutritious foods they know their children will eat to minimize the risk of wasting money (51, 52). Other members of the household were not the only external influence on purchasing patterns; the supermarket environment itself was also reported to play a detrimental role.

The cognitive burden associated with shopping for food in the supermarket context may be increased for some vulnerable groups, including those living on a low income. Supermarkets are busy environments, stocking and selling approximately 25,000–40,000 products (60, 61). Consumers are faced with a wide variety of stimuli during each shopping trip (62), including many aisles of food items that are promoted using various types of deals or offers (e.g., Buy One Get One Free, price matches, loyalty card prices), as well as other shoppers, bright lighting, and, in some retail outlets, in-store music. Consumers have finite mental resources and, therefore, attempting to determine the best food prices or identify products that best meet their nutritional needs within this busy store environment could result in cognitive overload (63). Even after devising meal plans, writing shopping lists, or having strong intentions to make healthier choices, cognitive overload has been shown to cause consumers to make less healthy purchasing decisions (64). The financial strain faced by those experiencing FI may further deplete available mental resources, resulting in an even greater cognitive load when shopping in the supermarket, potentially leading this group to be more likely to disengage with health-promoting behaviors (54, 65–67).

Within the supermarket space, tempting offers on less healthy products can lead to unintended purchases. To try and help stay on track and avoid *in-store temptation* and unintended purchases, participants utilized (often unsuccessful) strategies such as self-talk and listening to music. However, online shopping was regarded by some as a successful method of avoiding impulse buys of less healthy food items. The online supermarket space was perceived as having fewer distractions or temptations and as somewhere participants felt more in control of their purchasing decisions. Online consumers are not exposed to the full range of products and are able to store items in their favorites or re-add previously purchased items, saving time and money (68). Participants described using filters, available on the supermarket website, as a strategy to prevent them from seeing less healthy food items in the first place, contrasting this to the physical in-store experience, where such encounters were deemed inevitable. The potential positive impact of online supermarkets in terms of reducing impulse purchases, allowing for the allocation of more of the budget toward healthier products, and a higher nutrient density score and lower caloric density of baskets has been recognized previously (69, 70). However, concerns around the short shelf life of items delivered and the potential for food waste were described as barriers to engaging in online shopping practices by participants in the current study. Legislation such as the recent UK Government high fat, salt and sugar (HFSS) regulations, restricting the placement and promotion of HFSS products in prominent in-store and online locations, introduced in England in 2022 (71), may go some way to help mitigate in-store temptation and unintended purchases. However, HFSS foods are still available elsewhere in-store and such restrictions do not make healthier food items more affordable; therefore, this type of legislation seems unlikely to reduce existing dietary health inequalities (72).

In the face of a restricted income and the challenges outlined so far, it would be easy to comprehend how the environmental sustainability of the food purchased by PLWO and FI may be of little concern. Indeed, research shows that society views sustainability as a low-priority issue for those living on a tight budget who, it is argued, may have more immediate, pressing concerns, such as paying rent (73). People concerned about the environment are stereotypically perceived to be of White ethnicity, well-educated and moderately wealthy, however, the environmental concerns of ethnic minority and low income groups have been underestimated (74). Participants in the current study demonstrated a good understanding of, and a desire to, enact environmentally sustainable shopping practices (i.e., purchasing locally grown food items, items with recyclable packaging, plant-based meat alternatives), suggesting that the ability to purchase healthy, sustainable food is a priority not solely reserved for more affluent members of society. The high cost associated with environmentally sustainable foods presented a barrier to their purchase for almost all participants; however, during discussions, it became apparent that strategies enacted to help maximize limited food budgets resulted in an unintended consequence, that of *unconscious, environmentally sustainable shopping practices*. To lower GHGE and meet global emissions targets set by the Paris Agreement on climate change (75), people in high-consuming regions (the Americas, Europe, and Oceania) need to halve current levels of food waste (76). The notion of food as too precious to waste led participants to frequently purchase tinned and frozen fruits and vegetables as opposed to fresh alternatives, with a much shorter shelf life, reducing the potential for household food waste. Buying tinned and frozen fruit and vegetables was viewed as a necessity, a behavior participants would prefer not to enact due to perceptions of inferior nutritional quality (21, 54), but one their limited budget compelled them to make. Participants also talked about the struggle to purchase and consume meat as part of their diet. The World Cancer Research Fund recommends that individuals should reduce their consumption of red and processed meat to two servings or less per week by 2030 and 1.5 servings a week by 2050 (76). Our interviews and focus groups took place between June and December 2023, following a period of food and non-alcoholic beverage inflation that reached 19.2% in March 2023, the highest rate in over 45 years (77). As a result of the steep increase in food prices, participants had sought lower-cost meal alternatives with some describing replacing meat-based meals with vegetarian alternatives to keep their food costs manageable. Through necessity, we see how people living on a low income may already be unconsciously engaging in more environmentally sustainable food purchasing and consumption patterns. Although it is important to highlight, these practices are not made through choice. Such purchasing patterns do not mean the individual is buying and consuming a healthy diet in line with FBDGs. PLWO and FI should have the available resources to acquire the healthy, environmentally sustainable food they want to buy rather than being forced to make purchases that could be viewed as having some environmental benefits but may do little to support their physical and mental health.

## 5 Strengths and limitations

The current study had several strengths, including insights from hard-to-reach population groups, including people living on a low income and non-UK nationals. It is important to include the voices of these under-researched populations who are potentially at greater risk of experiencing FI. Furthermore, conducting the qualitative research as part of the FIO Food project allowed us to build on and contextualize prior quantitative research (42, 44). The interviews and focus groups provided the opportunity for a more in-depth investigation of the external influences and internally held views and beliefs that shape purchasing patterns and diet quality of PLWO and FI within the supermarket context.

The majority of participants self-reporting their health as good or fair, just over two-thirds indicated they lived with one or more health conditions (i.e., Type 2 diabetes, arthritis, and depression), which, if they have a substantial and long-term negative impact on daily living, can be considered a disability (78). However, the impact of these health conditions or a disability on shopping experiences, and vice versa, was not widely discussed. It is possible the impact of the participants’ health conditions was not great enough to be perceived as a disability or have an influence on or be influenced by their food shopping experience. On the other hand, a lack of discussion surrounding this issue may have been the result of the questions asked and a lack of focus on this specific topic. Alternatively, it is argued, constant repetition of an action within a specific context leads to this action occurring automatically whenever the contextual cue is encountered by the individual (79). Participants may have lived with their disability or health condition for a long period of time, and therefore, behaviors to mitigate any impacts within the supermarket context could have become habitual, meaning the participant could have failed to explicitly report on them. Discussions during the interviews may have focused on more salient influences of the present time.

## 6 Conclusion

This research focuses on the supermarket context, one small part of the complex, wider food system. However, with the majority of food consumed within the home purchased from this retail space, it undoubtedly plays an important role in shaping household food purchases. Within the supermarket, fluctuating price promotions, an increased number of deals and offers on less healthy food items, the overwhelming environment, and skepticism around the genuineness of deals and offers, all potentially hinder the purchase of healthy, environmentally sustainable foods for PLWO and FI. Such factors interact with broader issues such as a lack of adequate Government financial assistance (or the inability to access this support, as is the case for many non-UK nationals) alongside considerations such as the role of others in the household. PLWO and FI are forced to make difficult choices when shopping for healthy, environmentally sustainable food at the supermarket due to their limited budget, which can ultimately widen health inequalities between the most and least affluent within society. Understanding the interconnected challenges faced by this vulnerable group can help inform future research to guide the development of interventions and policies to adequately address and reduce health inequalities and socially patterned health outcomes currently present in high-income countries.

## Data Availability

The datasets presented in this article are not readily available because any requests will be reviewed on a case-by-case basis. Requests to access the datasets should be directed to Emma Hunter, e.hunter7@rgu.ac.uk.

## Footnotes

^1^ www.prolific.com
