# Supplementary material for: The people behind the pounds: a qualitative exploration of factors that help or hinder healthy, sustainable food purchases for people living with obesity and food insecurity in the UK
Source: Front Nutr. 2025 Oct 8;12:1646056. doi: 10.3389/fnut.2025.1646056 (PMC12540083; doi:10.3389/fnut.2025.1646056)
Supplement: Supplementary file 1 [file Table_1.DOCX]

Supplementary Data

Topic Guide Interviews

**Opening script**

**Introduction to the topic and instructions:**

Hello and welcome to our interview. Thank for agreeing to take part. My name is Emma and this is Flora and we’ll be facilitating the interview today. Before we start do you have any questions or concerns you’d like to raise that have occurred to you since you agreed to take part?

(Answer any questions)

Ok, great.

So, our research project is concerned with helping people who want to lose weight and who are living on a tight budget to shop more easily for the food items they believe will help them to do that. We are also interested to understand your views about sustainability, and how or if this issue fits in when you are shopping for food.

The interview should last about 30 minutes, but it depends how much or as little you feel you have to say about the things we will talk about today. You do not need to answer all the questions and if you don’t feel you have anything to say on a particular question, just let me know and I will move onto the next one. I’d also like to stress this not a test of knowledge (there are no wrong answers), we’re simply trying to find out your experiences and views on this topic and we are really grateful for the time you are giving and the insights you are sharing with us today. There will be time at the end where you can ask any questions you might have. We will be recording today’s discussion so we don’t miss anything important or have to write down everything you say. Your responses will be treated with full confidentiality, any potentially identifying information you share, such as names or places, will be removed so you can’t be identified. If you would like to take a break at any point, you can let me know and we can continue when you are ready. Just to let you know, you will be provided with a £25 shopping voucher as a thank you for taking part.

Do you have any questions before we start?

**Opening Question**

**Broad experience:**

So, thinking about those broad questions our project is focused on:

- **How would you describe your experience of shopping for food?**
  - *To further explain, ask:*

***Think about a usual trip to the supermarket, how does it feel when you are shopping?

- **Do you shop online as well as in store?** (or vice versa if they discuss online shopping first)
  - *If yes:*

*How do those experiences differ?

*Which do you prefer and why?

**Choice of retailer:**

Next we’d like to ask a few questions on where you typically shop:

- **Where would you say you normally do the majority of your shopping?**
  - *If they don’t already state reasons, ask*:

*What are the reasons, would you say, that means you normally shop in these places?

- - ***If they state more than one shop/ supermarket, explore****:*

*Can you tell us a little bit more about the types of things you buy at the different stores?

- (If not covered by above) **What are your experiences of shopping at X?**
  - *Explore likes and dislikes:*

*What do you like about shopping at X? What do you dislike?

- **Are there any places or shops that you would not use for food shopping?**
  - *If they don’t provide much information, explore:*

*Why do you avoid using those shops/ places?

- **Have other people in the supermarket, such as supermarket staff, check out operators, other shoppers had any influence on where you shop?**
  - *If yes, explore*:

*What influence have they had (i.e. shopping habits/ purchases made/ how often they attend/ length of time spent considering what to buy?)

**External influences impacting purchasing:**

Great, ok, so next we would like to ask about some things that might have an impact on how you shop and what you buy

- **Can I ask, who do you shop for?**
  - *If yes:*

*Who

*Do they go to the shop with you?

*How often do they go with you?

- **Do you think they have an influence on what you buy when shopping in store/ online?**
  - *If yes:*

*In what way?

- **Is there anything else that you think influences what you buy?**
  - *To probe further we can ask about:*

* Religion/ religious celebrations or culture

* Health conditions

- - Explore in what way these influence what they buy
- **Have you ever accessed or do you access emergency food provisions?**
  - *If yes and the participant is willing to talk about this*

* What kinds of food would you typically receive?

* How often would you receive this?

* Are you able to continue to shop at the supermarket during this time

* (if yes) what items do you continue to buy?

- **Are there any days in the week or times of day that you prefer to shop?**
  - *If yes:*

*Why is that?

- **Do you plan what you are going to buy ahead of time:**
  - *Can prompt:*

*Do you tend to make a shopping list?

*Plan your meals for the week?

*If shopping is mainly online we could probe:*

Do you store favourites/ re order same each week/ add to the basket throughout the week?

- **Do you find it easy to stick to your list?**
  - *Can probe:*

*Why

*Why not

- **Do you ever take advantage of supermarket promotional offers?**
  - *Can probe:*

*What type of offers do you tend to take advantage of?

*Why?

*If they indicate that they don’t, we can ask*

*Is there any reason you don’t take advantage of these offers?

**Food purchasing behaviour**:

Thank you. Ok, so next we’d like to discuss your shopping behaviour

- Firstly, **how often would you say you go food shopping?**
- **And thinking about the foods you typically buy, what leads you to buy these things?**

*If they struggle to think of reasons, could prompt:*

*Would you say you buy these things:

…out of habit?

…because you like the taste?

…for others in your household?

…because of the price/ good value for money?

…longevity/ shelf life/ because it won’t go out of date quickly?

…ease of prep and/ or cook?

…calorie content?

…dietary requirements?

…nutritional content?

…quality?

…freshness?

…satiety/ is filling/ fills you up for longer?)

- **Have your shopping habits changed recently or as a result of Covid or the cost of living crisis?**
  - *If yes:*

*In what way has this changed?

* Have you stuck with the changes you made during Covid?

*What products do you buy more/ less of and why?

- **Are there any foods would you like to buy that you don’t typically purchase?**
  - *If yes:*

*What types of foods would you like to purchase?

*What would be the main reasons for wanting to buy these foods?

*What is preventing you from buying them at the moment?

- **What kinds of things do you think supermarkets could do to help make buying healthy food easier?**

If needed, prompt:

- Price discount on healthy food products
- Nutrition shelf labelling (shelf signs identifying healthy food)
- Nutrition education in store (e.g., sample shopping lists, recipe suggestions)
- Increased stocking and availability of healthy food in supermarket
- Healthy food samples (e.g., aisle demonstrations, taste samples)
- Place healthy food in aisle endcaps (end of the aisle)

**Dietary changes**

As we mentioned, we’re keen to speak to people on a tight budget who are looking to lose weight and we wondered,

- **Have you made any changes to your diet to try and reduce your weight?**
  - *If yes:*

*What changes have you made?

- - *If no:*

*What changes would you like to make?

- **Are you able/ do you think you will be able to buy the food you need to help you reduce your weight?**
  - *If yes:*

*What has been your experience of buying these foods?

Could prompt:

…Spending more money

…Spending the same amount of money

…Spending more time in store

…Spending less time in store

…Opinion of others in household

- - *If they would like to buy but don’t yet do so:*

* What food would you like to buy to help you reduce your weight?

* What are the barriers/ what stops you from buying these foods?

* What could the supermarket do to help you buy these foods?

* Is there anything else you think could help you buy these foods?

**Sustainability**

We realise it can be a struggle to buy healthy, good quality food and the FIO Food study is looking at ways we can help support people buy the foods they would like to so they can eat well, improve their health and reduce their weight. The FIO Food Study is also interested in the environmental aspect, the sustainability or the impact of our diet on the planet. I’m not sure if this is something you have thought about before and it’s completely fine if you haven’t but I’d like to ask a few questions on sustainability.

- **When I say sustainable foods or food sustainability, what does that mean to you?**
- *If required, we can offer examples:*

…Greenhouse gas emissions

…Land use

…Water use

…Buying local produce

- **Is the environmental impact of the food you buy something you tend to think about when you are shopping?**
  - *If yes:*

*In what way does this influence what you buy or eat?

*How do you find information on the sustainability of the food you buy?

- - *If no:*

*Would you like to make more environmentally sustainable food purchases or meals? *If yes:*

*What sort of changes might you like to make

Could probe:

…meals with less meat

…meat free meals

…bulking out meals with beans, peas, lentils or other veg

*If no:*

*Would you be willing to share your reasons for not wating to make these changes?

*Do you ever notice anything in store/online/on product about sustainability?

- **Do you think the supermarkets could do anything to make buying environmentally sustainable food easier?**

*If yes:*

* What could they do?

*Do you ever notice anything in store/online/on product about sustainability?

*Do you feel supermarkets could do anything to help you make these types of purchases?

Could prompt

- Price discount on sustainable food products
- Labelling (sustainably sourced, locally grown)
- Recyclable/reusable packaging
- Sustainability education in store (e.g., sample shopping lists, recipe suggestions)
- Increased stocking and availability of sustainable food in supermarket
- Sustainable food samples (e.g., aisle demonstrations, taste samples)
- Place sustainable food in aisle endcaps (end of the aisle)

**Concluding the session:**

Great, that’s all the questions I have today.

Is there anything else you would like to add?

Ok, that’s us at the end of the interview. Do you have any other questions before we finish? (Answer any questions)

You have our details so if you do think of anything, please get in touch.

Your contribution today is greatly appreciated and we would just like to take this opportunity to thank you for taking part.
